# Concurrent robotic colorectal surgical oncology training within a structured mentored international fellowship program

**DOI:** 10.1007/s11701-025-02287-4

**Published:** 2025-03-27

**Authors:** Trevor M. Yeung, Philip Bauer, Ramy Behman, Andrea Marcadis, Adam Studniarek, Garrett Nash, Julio Garcia-Aguilar

**Affiliations:** 1 Department of Colorectal Surgery, Memorial Sloan Kettering Cancer Center, 1275 York Avenue, New York, NY 10065 USA; 2 Division of Colorectal Surgery, Department of Surgery, The Chinese University of Hong Kong, Prince of Wales Hospital, Sha Tin, NT Hong Kong; 3 Department of Colorectal Surgery, Weill Cornell Medical Center, 525 E 68 St, New York, NY 10065 USA

**Keywords:** Robotic colorectal surgery, Training, Fellowship

## Abstract

Robotic colorectal surgery is increasingly adopted worldwide, with mentored programs for established surgeons becoming more common. However, there is a paucity of dedicated robotic training programs for colorectal fellows. This study aims to assess the feasibility and efficacy of a structured, apprentice-based robotic colorectal training program delivered to multiple fellows concurrently. The fellowship program incorporates simulation training, dry/wet laboratory work, dedicated robotic console time in the operating room (OR) and individualised mentorship. Overall robotic proficiency was assessed using the Global Evaluative Assessment of Robotic Skills (GEARS) and procedure-specific proficiency was assessed using a modified European Academy of Robotic Colorectal Surgery (EARCS) Global Assessment Score (GAS) throughout the fellowship. A total of 59 cases (29 right hemicolectomies, 30 anterior resections) were evaluated between August 2023 and July 2024. Significant improvements were observed in GEARS scores (*p* = 0.0065) and modified GAS for both right hemicolectomies (*p* = 0.0052) and anterior resections (*p* = 0.0005), demonstrating a high level of competence and independence. Mean operative times were 213 min (right hemicolectomy) and 328 min (anterior resection). Median length of stay in the hospital was 2 days (right hemicolectomy) and 4 days (anterior resection). Median lymph node yield was 29 (right hemicolectomy) and 26 (anterior resection). There was 0% involved margins for both procedures. Robotic colorectal surgical oncology training delivered to multiple fellows concurrently in an apprenticeship model with dedicated console time is achievable and successful, leading to high levels of robotic competency and independence, whilst maintaining a high standard of clinical care and oncological outcome.

## Introduction

Robotic colorectal surgery is increasingly being implemented globally [1–3]. Whilst robotic mentored programs exist for established colorectal surgeons [4–7], there are far fewer dedicated robotic programs for colorectal fellows [8–10]. Challenges and questions remain on how to best implement training programs that would provide sufficient exposure and standardised assessment resulting in full competency upon completion of these training programs [9, 11, 12].

Since 2014, Memorial Sloan Kettering Cancer Center (MSKCC) has offered robotic surgical training as a critical component of an international advanced colorectal oncology fellowship program; 42 fellows have successfully completed this robotic surgical training in this setting. All fellows complete a program of surgical simulation and dry/wet laboratory work before commencing their robotic surgical training in the operating room. Each case is staffed bedside by a robotic physician assistant or surgical resident, allowing the fellow to have dedicated console time. Robotic training is delivered in a structured and mentored fashion, tailored to the individual needs and requirements for each fellow’s development.

The aims of this study are to demonstrate the feasibility of delivering a structured and apprentice-based robotic colorectal training program for multiple fellows concurrently and to assess the progression of surgical fellows undertaking this training program.

## Methods

This was a prospective study undertaken between August 2023 and July 2024. All patients undergoing a robotic colorectal resection as part of the fellowship training program were included. Ethical approval was waivered by the MSKCC institutional board.

### MSKCC robotic training program

During 2023–2024, there were 3 full-time colorectal robotic fellows based at MSKCC (2 international, 1 US-based), who have previously completed colorectal surgical training. The one-year robotic fellowship follows an apprenticeship model, in which each fellow rotates for a month with a different attending. In total, there are 9 clinical months on service, and 3 elective months, during which time the fellows are free to choose to engage in research projects and/or join other specialties for further clinical experience. Additionally, there were two trainees from a combined Accreditation Council for Graduate Medical Education (ACGME)-sponsored New York Presbyterian (NYP)-Cornell/MSKCC colorectal training program; each spent 6 months on clinical service at MSKCC. After initial orientation for subsequent operations, the bedside is staffed by a dedicated robotic colorectal physician’s assistant (PA) or surgical resident, allowing the fellow to spend dedicated training time on the console.

### MSKCC robotic training pathway

At MSKCC, the robotic platform used is the da Vinci Xi (Intuitive Surgical, Ltd., Sunnyvale, USA) dual console system. The dedicated robotic training pathway is split into 3 components – orientation, surgical, and certification.

The orientation component is led by the lead robotic colorectal physician assistant (PA). Prior to starting the program, all colorectal fellows must complete the online robotic training at http://www.davincisurgerycommunity.com (P8 version). They then attend a dry lab workshop focusing on docking, port insertion, instrument exchange, bedside assistance, robotic suturing, robotic stapling and console training. The fellow then must complete a series of designated modules on the robotic simulator with a minimum score of 85% in each module (see Appendix 1). Upon successful completion of these modules, the fellow then attends a dedicated hands-on wet laboratory workshop covering tasks that include tissue dissection, vessel sealing, suturing, stapling, intracorporeal anastomosis using porcine tissue models.

The surgical component is led by surgical attendings. At the start of each new robotic surgical rotation, the fellow participates in an update session to review the safe use of the equipment. The fellow reviews a series of video robotic cases specific to the procedures being performed. Annotated videos are available on the departmental computer shared drive, listed under each different attending. Training on the console is component-based, specific feedback is given intra-operatively, and debriefing is performed at the end of each case to highlight areas for improvement. Video recordings of the cases are reviewed to aid feedback and identify key steps to focus on for subsequent cases.

The certification of robotic surgical competency is granted at the end of the fellowship after satisfactory completion of the aforementioned components of the robotic training pathway as well as summative assessments by attendings.

### Robotic proficiency assessment

Robotic proficiency was assessed using two standardised scoring systems:

(A) Global Evaluative Assessment of Robotic Skills (GEARS).
  At the start and end of the training program, all fellows used the Global Evaluative Assessment of Robotic Skills (GEARS) score to assess overall robotic proficiency [13]. This is a validated assessment tool developed by deconstructing the fundamental elements of robotic surgical procedures. It is composed of six domains: depth perception, bimanual dexterity, efficiency, force sensitivity, autonomy and robotic control. Proficiency in each domain is scored on a 5-point Likert Scale, with an overall performance score ranging from 6 to 30. Higher scores equate to better performance.
(B) European Academy of Robotic Colorectal Surgery (EARCS) Global Assessment Score (GAS).
  To assess procedure-specific skills, all fellows used a modified version of the EARCS Global Assessment Score (GAS) form [14]. For right hemicolectomies, modules on colonic resection and colonic intracorporeal anastomosis were assessed (Appendix 2). For anterior resections, modules on colonic resection, TME dissection and intracorporeal formation of the rectal anastomosis were assessed (Appendix 3). Each module consists of several component steps. Each component is scored from 1 to 6 (or not applicable if the step is not performed by the fellow). Higher scores equate to a higher competence level for each component.
  The GAS was compiled at 2 monthly intervals for both right hemicolectomies and anterior resections. If a particular component was not performed by the fellow during that case, the fellow was asked to look back over the preceding cases performed during those 2 months and to score that component at the level they felt best correlated with their skills level at that stage. This meant that the GAS score was not unfairly penalised if the fellow did not perform all components of that case, and that the GAS score was a fair representation of the fellow’s skills set at that stage of training.

### Statistics

Data were analysed using GraphPad (http://www.graphpad.com) and Microsoft Excel for Microsoft 365. For patient characteristics and perioperative/postoperative outcome measures, parametric data was expressed as mean with standard deviation and non-parametric data was expressed as median with interquartile range. Categorical variables were expressed as a number and percentage. The paired *t*-test was used to compare differences in mean scores of robotic proficiencies between the two groups. A significance level of *p* < 0.05 was considered statistically significant.

## Results

### Patient characteristics and operative outcomes

The fellows that participated in this study assessed their progress at 2 monthly intervals during their fellowship. A total of 59 cases were scored, 29 right hemicolectomies and 30 anterior resections. Baseline patient characteristics are shown in Tables 1 and 2. Perioperative and postoperative outcomes are summarised in Tables 3 and 4.

**Table 1:** Baseline patient characteristics for robotic right hemicolectomies

	Total *n* = 29
Sex	
Male	15 (51.7%)
Female	14 (48.3%)
Median age (IQR)	62 (54–75)
Mean BMI (SD)	28.5 (4.1)
Location of tumor*	
Terminal Ileum	2 (6.9%)
Appendix	1 (3.4%)
Cecum	10 (34.5%)
Ascending	9 (31.0%)
Hepatic	4 (13.8%)
Transverse	4 (13.8%)

*Total more than 100% as one patient had both ascending

and transverse colon CA

*IQR* Interquartile Range

*SD* Standard Deviation

**Table 2:** Baseline patient characteristics for robotic anterior resections

	Total *n* = 30
Sex	
Male	17 (56.7%)
Female	13 (43.3%)
Median age (IQR)	55 (42–64)
Mean BMI (SD)	30 (4.9)
Location of rectal tumor	
Low < 5 cm)	3 (10.0%)
Mid (5–10 cm)	10 (33.3%)
High (> 10 cm)	17 (56.7%)
Neoadjuvant therapy	
Yes	18 (60.0%)
No	12 (40.0%)
Pre-operative radiological staging (MRI and CT)	
Tx	4 (13.3%)
T1	3 (10.0%)
T2	2 (6.7%)
T3	20 (66.7%)
T4	1 (3.3%)
CRM threatened per preop MRI	
Yes	5 (16.7%)
No	25 (83.3%)

*IQR* Interquartile Range

*SD* Standard Deviation

**Table 3:** Perioperative and postoperative outcomes for robotic right hemicolectomies

	Total *n* = 29
Procedure	
Right hemicolectomy	27 (93.1%)
Extended right hemicolectomy	2 (6.9%)
Mean operative time, min (SD)	213 (64)
Median blood loss, mL (IQR)	50 (25–50)
Conversion to open	
	1 (3.4%)
Intraoperative complications	0 (0%)
Postoperative complications	6 (20.7%)
Clavien Dindo 1	3 (10.3%)
Clavien Dindo 2	2 (6.9%)
Clavien Dindo 3a*	1 (3.4%)
Tumor margin involved	0 (0%)
Median lymph node yield (IQR)	29 (21–42)
Median length of stay, days (IQR)	2 (2–4)
30-day reoperation	0 (0%)
30-day readmission	0 (0%)
Final Pathology	
pT0	1 (3.4%)
pT1	6 (20.7%)
pT2	5 (17.2%)
pT3	13 (44.8%)
pT4	3 (10.3%)
Benign	1 (3.4%)
pN0	19 (65.5%)
pN1	5 (17.2%)
pN2	4 (13.8%)

*Patient had hematochezia post-operatively. Colonoscopy was performed and a visible bleeding vessel was identified at anastomosis which was clipped. Patient did not require any blood transfusion

*IQR* Interquartile Range

*SD* Standard Deviation

**Table 4:** Perioperative and postoperative outcomes for robotic anterior resections

	Total *n* = 30
Procedure	
Anterior resection without ileostomy	19 (63.3%)
Anterior resection with ileostomy	11 (36.7%)
Mean operative time, min (SD)	328 (102)
Median blood loss, mL (IQR)	50 (50–50)
Conversion to open	0 (0%)
Intraoperative complications	0 (0%)
Postoperative complications	8 (26.7%)
Clavien Dindo 1	5 (16.7%)
Clavien Dindo 2	3 (10.0%)
Tumor margin involved	0 (0%)
Median lymph node yield (IQR)	26 (19–35)
Median length of stay, days (IQR)	4 (3–6)
30-day reoperation	0 (0%)
30-day readmission	1 (3.3%)*
Final Pathology	
pT0	4 (13.3%)
pTis	1 (3.3%)
pT1	3 (10.0%)
pT2	5 (16.7%)
pT3	13 (43.3%)
pT4	3 (10.0%)
No residual NET identified	1 (3.3%)
pN0	21 (70.0%)
pN1	4 (13.3%)
pN2	4 (13.3%)

*One patient represented to urgent care center with abdominal pain, CT was normal, patient was treated for UTI

*IQR* Interquartile Range

*SD* Standard Deviation

In the right hemicolectomy group, 15 patients (51.7%) were male, with a median age of 62 and a mean BMI of 28.5 (Table 1). Two patients (6.9%) underwent extended right hemicolectomy resections (Table 3). The mean operative time was 213 min with a median blood loss of 50 mL. There was 1 (3.4%) conversion to open, as the tumour appeared to involve the duodenum; however upon further assessment after open conversion, the tumor was freed from the retroperitoneum and no duodenal resection was required. There were no intraoperative complications. Six patients (20.7%) developed postoperative complications, 3 (10.3%) were Clavien Dindo (CD) grade 1, 2 (6.9%) were CD grade 2 and 1 (3.4%) was CD grade 3a. The anastomotic leak rate was 0%. The median length of stay was 2 days, and there were no 30-day readmissions nor reoperations. There were no positive resection margins in any specimen. The median lymph node yield was 29 (Interquartile range IQR 21–42).

In the anterior resection group, 17 patients (56.7%) were male, with a median age of 55 and a mean BMI of 30 (Table 2). 17 patients (56.7%) had a high rectal tumor (> 10 cm from the anal verge), 10 (33.3%) had a mid-rectal tumor (5–10 cm) and 3 (10.0%) had a low rectal tumor (< 5 cm). 18 patients (60%) had neoadjuvant therapy and 5 (16.7%) had a threatened circumferential resection margin (CRM). Out of the 30 anterior resections, 11 patients (36.7%) had a diverting loop ileostomy formed at the same time (Table 4). The mean operative time was 328 min with a median blood loss of 50 mL. There were no conversions to open and no intraoperative complications. 8 patients (26.7%) developed postoperative complications, 5 (16.7%) were CD grade 1, and 3 (10%) were CD grade 2. The anastomotic leak rate was 0%. The median length of stay was 4 days, and there were no 30-day reoperations. There was 1 (3.3%) 30-day readmission for a patient with abdominal pain, whose CT abdomen and pelvis was negative and was treated for a urinary tract infection. There were no tumor margins involved in any specimen. The median lymph node yield was 26 (IQR 19–35).

### Training assessment and outcomes

**Fig. 1:**
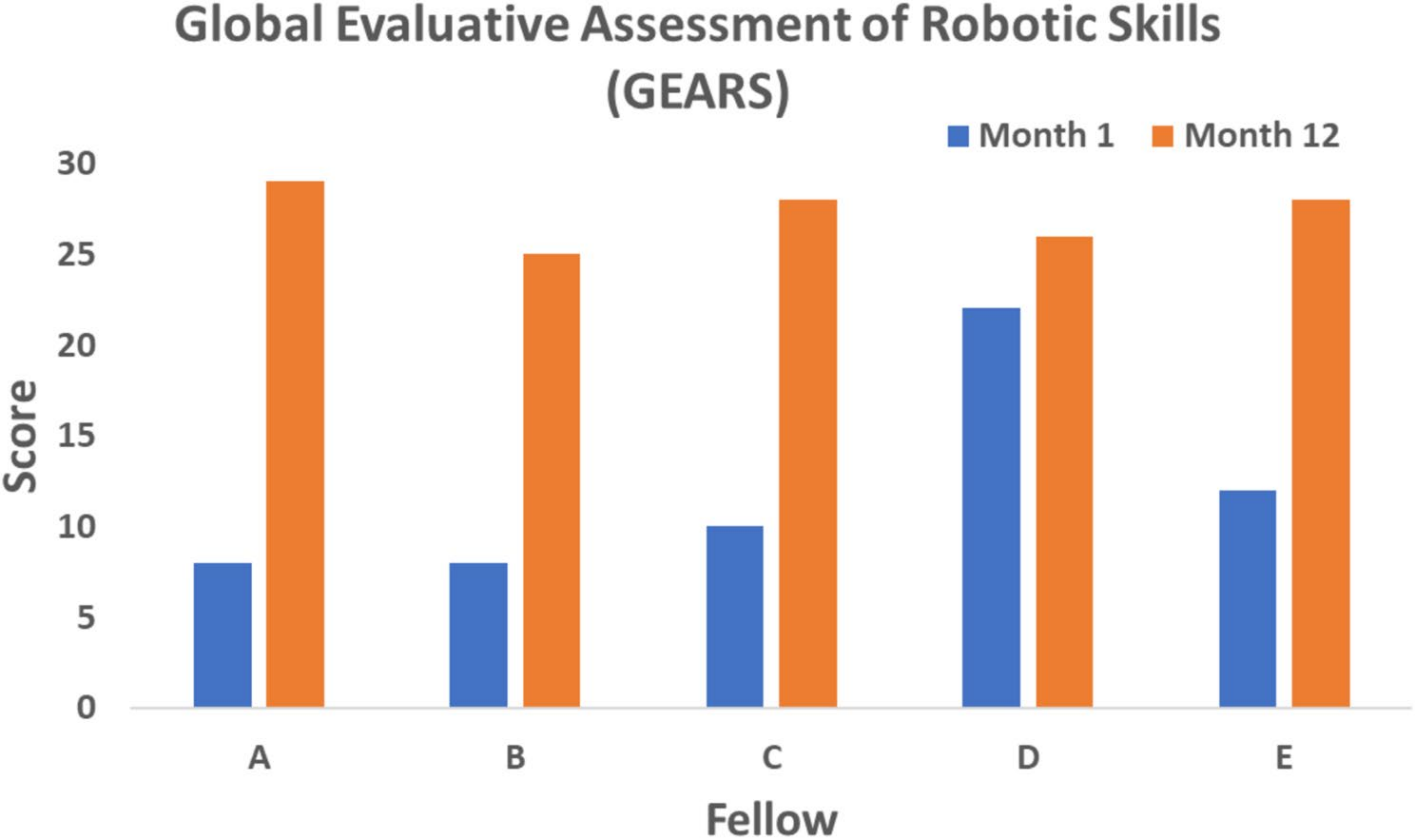
Global Evaluative Assessment of Robotic Skills (GEARS). Each fellow was asked to self-assess their robotic skills at the beginning and at the end of the fellowship in six domains: depth perception, bimanual dexterity, efficiency, force sensitivity, autonomy and robotic control. Proficiency in each domain was scored on a 5-point Likert Scale, with an overall performance score ranging from 6 to 30. Scores from first month were compared with the scores from the last month. Paired *t*-test, *p* = 0.0065

**Fig. 2:**
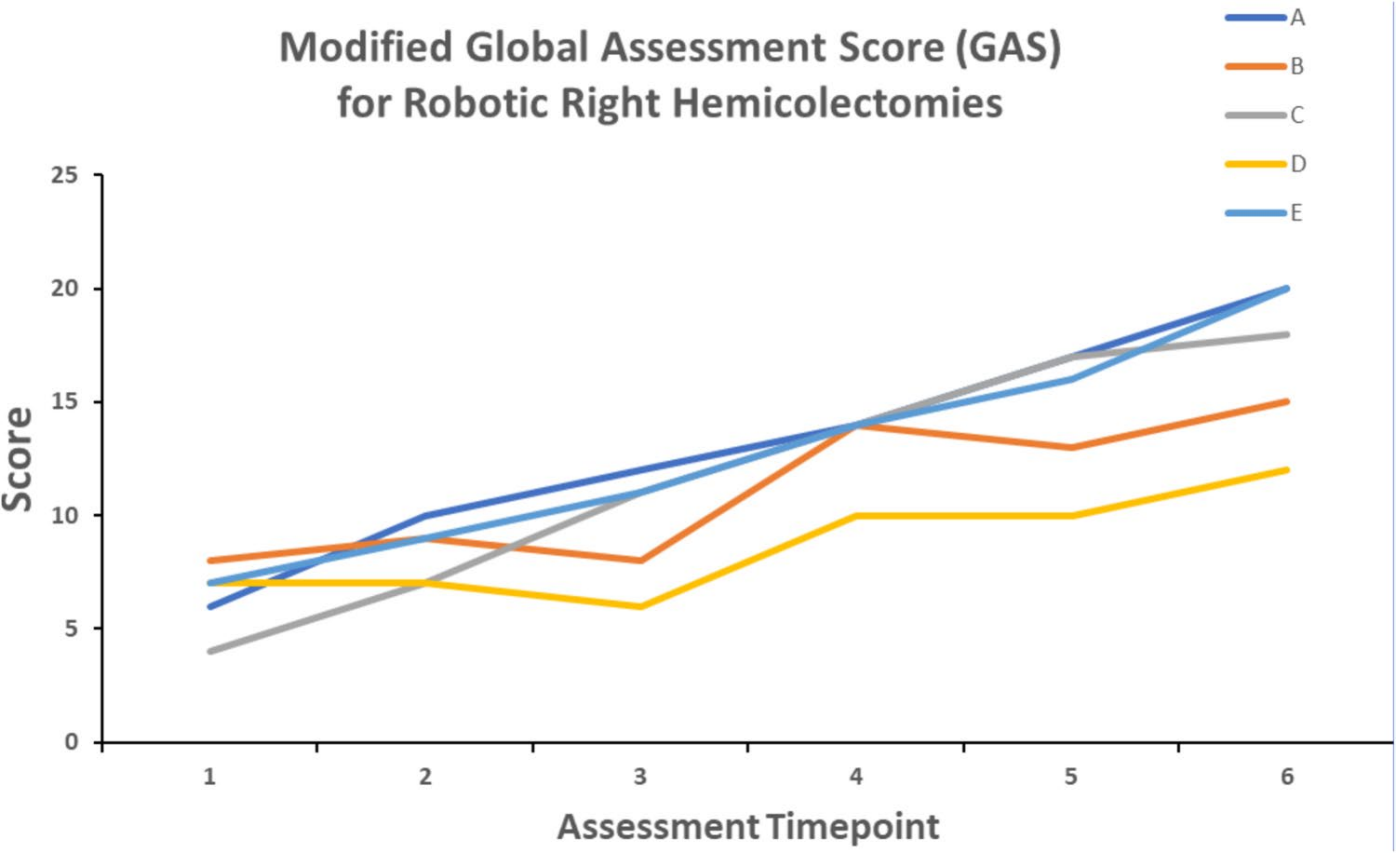
Modified Global Assessment Score (GAS) for Robotic Right Hemicolectomies. Every two months, each fellow assessed their proficiency in performing a robotic right hemicolectomy. The maximum score is 24 and a score of 20 or above represents independent and competent performance. Scores from the first two-month period were compared with the final two-month period. Paired *t*-test, *p* = 0.0052

**Fig. 3:**
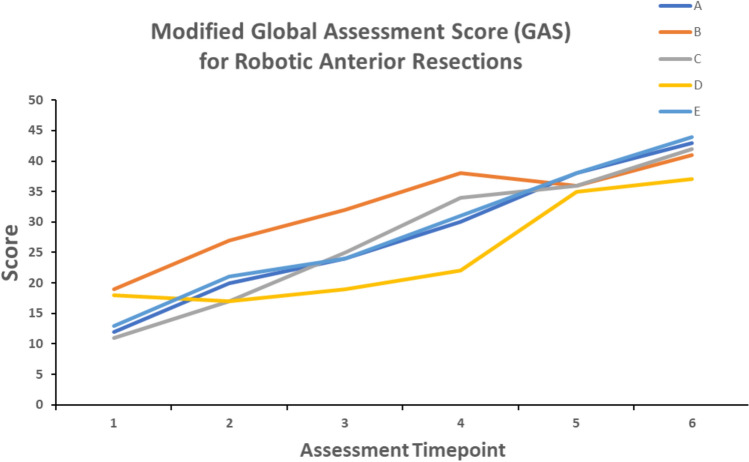
Modified Global Assessment Score (GAS) for Robotic anterior resections. Every two months, each fellow assessed their proficiency in performing a robotic anterior resection. The maximum score is 54 and a score of 45 or above represents independent and competent performance. Scores from the first two-month period were compared with the final two-month period. Paired *t*-test, *p* = 0.0005

Prior to this fellowship, two fellows had no previous experience with the robot console, one fellow had limited experience in robotic colorectal surgery (10 console cases) and two fellows had already received their Da Vinci certificate of training equivalency at other institutions. All fellows had significant improvements in their GEARS score (Fig. 1), with a mean overall score of 12 in the first month and 27.2 in the last month, *p* = 0.0065. For the modified GAS, there was significant improvement over the course of the fellowship for all fellows for both robotic right hemicolectomies (*p* = 0.0052, Fig. 2) and robotic anterior resections (*p* = 0.0005, Fig. 3), with overall scores commensurate with attaining high levels of competence and independence in robotic colorectal surgery.

Over the course of the year, the remaining three fellows obtained the Da Vinci Certificate of Training Equivalency from Intuitive Surgical. Overall, during the fellowship, the fellows each performed between 64 and 98 robotic operations as primary console operator, including robotic right colectomies (range 13–26), robotic high anterior resections (range 13–17) and robotic low anterior resections (range 23–30).

## Discussion

This study demonstrates that robotic colorectal surgical oncology training delivered to multiple fellows concurrently in an apprenticeship model is achievable and successful, whilst maintaining a high standard of clinical and oncological care. This is supported by the significant improvement in GEARS score by all fellows, and the significant increase in the modified GAS for both robotic right hemicolectomies and robotic anterior resections. Towards the end of the 12-month training period, fellows were attaining high GAS which correlates with a high degree of competence and independence achieved in robotic colorectal surgery. Out of the five fellows who completed the program last year, four have been appointed at the level of attending in their new institutions and continue with robotic colorectal practice and one has continued with their robotic training at MSKCC as a fellow.

This study supports the implementation of component-based training within a formal and structured curriculum, incorporating simulation, laboratory work, standardized steps and regular feedback on progress to achieve a successful surgical robotic training program [15–19]. One of the key drivers of success in our fellowship program is our ability to provide dedicated console time for fellows. This has been possible through the support network of (i) advanced practice providers/nurse practitioners who are able to cover clinical duties on the floor [20], thereby freeing up the fellow to go to OR and (ii) dedicated robotic colorectal PAs who assist bedside in the OR, allowing the fellow more time to be trained on the console. This is entirely consistent with a study that demonstrated that having a dedicated robotic PA bedside increased trainee console time in general thoracic surgery by almost double (45.8 min vs 80.9 min), and increased the average portion of a case performed by a trainee from 28% to 77.1% [21].

One of the unique features of our fellowship program is that it allows for multiple fellows to be trained concurrently. Our program is based on an apprenticeship model, where each fellow is assigned to a different attending (or two attendings) per month. As our institution has the resources to run and staff multiple robotic OR lists per day, this means that each fellow will typically have access to 2–4 OR robotic operations a week. The provision of a dedicated mentor each month allows the fellow to rapidly improve and develop their robotic surgical skills. We feel that the requirement for all fellows in our program to complete surgical simulation modules [9] and undertake dry and wet laboratory work [22] before starting on the console has really allowed the fellows to make the best use of personalised mentorship within the OR. Furthermore, fellows are encouraged to keep using the simulator [23] and review robotic videos [24] on the shared drive throughout the year to advance their skills whenever they are not in the OR. Even though two of the fellows had already acquired a certificate of equivalency from Intuitive prior to starting the fellowship, they continued to significantly improve their GEARS score and GAS, as they were exposed to more complex anatomical dissections and procedures at this institution e.g. complete mesocolic excision (CME), intracorporeal anastomosis.

The clinical implications of our study are that training fellows concurrently to a high degree of competency on the robotic console does not appear to compromise clinical care or oncological outcomes. Even with dedicated console time for fellows, operations can be completed in a timely fashion (mean operative time: right hemicolectomy 213 min, anterior resection 328 min). It is worth noting that robotic right hemicolectomies performed by fellows at our institution incorporate a complete mesocolic excision (CME) and the longer times reported in this study for a robotic right hemicolectomy reflect the additional complexities of this procedure. Patient outcomes (median length of stay in hospital: right hemicolectomy 2 days, anterior resection 4 days) and short-term oncological outcomes (median lymph node yield: right hemicolectomy 29, anterior resection 26; 0% positive margins for both procedures) were maintained at a high standard. Our results are consistent with reports of other training programs where the surgical and short-term oncological outcomes are similar between training cases and non-training cases [5, 8, 25].

The key strengths of this study are that it has been undertaken in a high-volume robotic colorectal cancer center, with a well-established and long-running advanced colorectal oncology fellowship program, welcoming both U.S.-trained and international-trained fellows, and that regular assessments were performed throughout the year for both right hemicolectomies and anterior resections. We recognise that the qualities that make this training program so successful (e.g. having a dedicated team of robotic PAs, significant resources allowing multiple robotic OR operations per day, nine colorectal surgical attendings with expertise in high volume robotic surgery) may not be immediately replicable in all other institutions. However, the principles of ensuring protected console time for fellows in the OR and dedicated mentorship are important and can be applied to other training programs. Another limitation of this study is that each GAS assessment represents only a snapshot of a fellow’s competency in time. Logistically, due to the high number of cases performed by each fellow, it was not feasible to perform a GAS on every case. However, we have mitigated this by using the modified GAS. If there was any component that was not performed by the fellow during a particular case, the fellow was asked to score that component at the level they felt best correlated with their skills level at that stage, resulting in a fairer overall representation of their ability. The fact that the fellows were able to complete GAS regularly throughout their fellowship gives a unique insight into their progress contemporaneously.

## Conclusion

Robotic colorectal surgical oncology training delivered to multiple fellows concurrently in an apprenticeship model with dedicated console time is achievable and successful, leading to high levels of robotic competency and independence, whilst maintaining a high standard of clinical care and oncological outcome.

## Data Availability

No datasets were generated or analysed during the current study.

## Appendix 1

List of simulation training modules which required completion with a minimum score of 85% for each module.

1. Needle targeting (needle driving)
2. Thread the rings (needle driving)
3. Energy switching 1(energy and dissection)
4. Energy dissection 2 (energy and dissection)
5. Suture sponge 2 (needle driving)
6. 30 - degree scope swap (camera control)
7. Three arm relay 2 (endowrist manipulation)
8. Ring walk 3 (camera control)

## Appendix 2

**Table:**

Modified Global Assessment Score (GAS) for Robotic Right Hemicolectomy	
Module: Colonic Dissection	
Transection of Vascular Pedicle	1 2 3 4 5 6 N/A
Mobilisation of Colon (Medial and Lateral)	1 2 3 4 5 6 N/A
Hepatic Flexure Mobilisation	1 2 3 4 5 6 N/A
Module: Anastomosis	
Intracorporeal Anastomosis	1 2 3 4 5 6 N/A
Scoring	
1—Step done by trainer/other trainee/not required in this procedure	
2—Physical input by trainer required (partly performed by trainer)	
3—Substantial verbal input by trainer required	
4—Only minor verbal input by trainer required	
5—Independent and competent performance, no substantial trainer input	
6—Masterful performance, couldn't be better	
N/A – Not applicable	
Maximum Score = 24	
Score of 20 or above represents independent and competent performance	

## Appendix 3

**Table:**

Modified Global Assessment Score (GAS) for Robotic Anterior Resection	
Module: Colonic Dissection	
Transection of Vascular Pedicle	1 2 3 4 5 6 N/A
Mobilisation of Colon (Medial and Lateral)	1 2 3 4 5 6 N/A
Splenic Flexure Mobilisation	1 2 3 4 5 6 N/A
Module: Total Mesorectal Excision	
Posterior Plane	1 2 3 4 5 6 N/A
Anterior Plane	1 2 3 4 5 6 N/A
Lateral Dissection	1 2 3 4 5 6 N/A
Low Pelvic Dissections	1 2 3 4 5 6 N/A
Transection of Rectum	1 2 3 4 5 6 N/A
Module: Anastomosis	
Intracorporeal Anastomosis	1 2 3 4 5 6 N/A
Scoring	
1—Step done by trainer/other trainee/not required in this procedure	
2—Physical input by trainer required (partly performed by trainer)	
3—Substantial verbal input by trainer required	
4—Only minor verbal input by trainer required	
5—Independent and competent performance, no substantial trainer input	
6—Masterful performance, couldn't be better	
N/A – Not applicable	
Maximum Score 54	
Score of 45 or above represents independent and competent performance	
